# Involvement of P2X7 receptors in chronic pain disorders

**DOI:** 10.1007/s11302-021-09796-5

**Published:** 2021-11-20

**Authors:** Wen-Jing Ren, Peter Illes

**Affiliations:** 1 School of Acupuncture and Tuina, Chengdu University of Traditional Chinese Medicine, Chengdu, 610075 China; 2 Rudolf Boehm Institute for Pharmacology and Toxicology, University of Leipzig, 04109 Leipzig, Germany

**Keywords:** Extracellular ATP, P2X7 receptor, Microglia, Astrocyte, Neuropathic pain, Inflammatory pain

## Abstract

Chronic pain is caused by cellular damage with an obligatory inflammatory component. In response to noxious stimuli, high levels of ATP leave according to their concentration gradient, the intracellular space through discontinuities generated in the plasma membrane or diffusion through pannexin-1 hemichannels, and activate P2X7Rs localized at peripheral and central immune cells. Because of the involvement of P2X7Rs in immune functions and especially the initiation of macrophage/microglial and astrocytic secretion of cytokines, chemokines, prostaglandins, proteases, reactive oxygen, and nitrogen species as well as the excitotoxic glutamate/ATP, this receptor type has a key role in chronic pain processes. Microglia are equipped with a battery of pattern recognition receptors that detect pathogen-associated molecular patterns (PAMPs) such as lipopolysaccharide (LPS) from bacterial infections or danger associated molecular patterns (DAMPs) such as ATP. The co-stimulation of these receptors leads to the activation of the NLRP3 inflammasome and interleukin-1β (IL-1β) release. In the present review, we invite you to a journey through inflammatory and neuropathic pain, primary headache, and regulation of morphine analgesic tolerance, in the pathophysiology of which P2X7Rs are centrally involved. P2X7R bearing microglia and astrocyte-like cells playing eminent roles in chronic pain will be also discussed.

## The P2X7 receptor

The human (h) *P2RX7* gene (R stands for receptor) is located on chromosome 12 and encodes 13 exons that translate into a 595 amino acid protein. A number of *P2RX7* isoforms derived from alternative splicing were identified both in humans and in rodents [1, 2]. Some variants are expressed and functional, for example, the human P2X7B receptor and the mouse and rat P2X7R variant k [3]. Several non-synonymous, intronic, or missense small nucleotide polymorphisms (SNPs) have been reported in the *hP2RX7* gene, expressing both gain-of-function and loss-of-function receptors.

Ionotropic P2X7 receptors are members of the P2X purinoceptor family [4, 5], which are located in the plasma membrane of various cell types and upon activation by high concentrations of ATP allow the inward flux of Na^+^/Ca^2+^ and the outward flux of K^+^, thereby inducing depolarization [6, 7]. P2XRs in general have a relatively simple structure consisting of assemblies of three identical linear protein subunits. These receptor subunits have N- and C-terminal intracellular tails, two transmembrane areas, and a large extracellular loop; the agonist-binding pouch of the receptor is located at the intersection of two neighboring subunits [8, 9].

The partial structure of the panda P2X7R [10] and later the full-length structure of the rat P2X7R [9] have been resolved, thereby identifying the kinetic changes occurring in the receptor structure after ATP-binding and the subsequent opening of the ion permeation pathway. The C-terminus of the receptor plays an important role in trafficking to the cell membrane; it also regulates receptor function (including signaling pathways), protein–protein interactions, and post-translational modifications [11–13]. Similar not only to P2X2 or P2X4Rs [14, 15] but also to transient receptor vanilloid channel 1 (TRPV1; [16, 17]) and acid-sensing ion channels (ASICs; [18, 19]), long-lasting occupation of the respective agonist-binding pouches was thought for a couple of years to result in a time-dependent dilation of the channels to constitute a pore through which molecules previously not passing the cell membrane barrier may diffuse into either direction.

With respect to the above reported channel dilations, the interpretation of these early whole-cell recording patch-clamp data obtained by reversal potential measurements turned out to be misleading [20, 21]. Participation of associated channel-forming proteins has been implicated in diffusion of large cationic molecules through P2X7Rs (e.g., pannexin-1; [22]), but convincing evidence now supports the view that the P2X7R by itself is endowed with the ability to conduct large organic cations, although at a slower pace than to conduct the smaller cations Na^+^, K^+^, and Ca^2^ [7, 23].

## P2X7 receptors at immune cells

ATP released from all types of damaged tissue via Panx-1 hemichannels or simply via the discontinuous cell membrane leads to stimulation of P2X7Rs and is associated with the activation of T-lymphocytes, and the differentiation of inflammatory T-helper lymphocytes (Th17) [12, 24, 25]. P2X7Rs also promote the chemotaxis of myeloid cells including macrophages, neutrophils, and the recruitment and activation of dendritic cells [26, 27]. The P2X7R participates not only in the secretion of the key inflammatory protein interleukin-1β (IL-1β; see below) but also in that of other interleukins and cytokines (IL-6, IL-8, IL-18, tumor necrosis factor-α (TNF-α)) and causes the release of chemokines (CCL2, CCL3, CCL7) and prostaglandin E2 (PGE2) [12, 25, 28, 29].

In the CNS, the expression of P2X7R mRNA and protein is highest in microglia, with much lower quantities present in neuroglial cells, astrocytes, and oligodendrocytes [30, 31]. Microglia are resident macrophages and the most important effectors of the brain’s innate immunity [32, 33]. Neuroglial cells assure the homeostasis of the brain (astrocytes) or generate the myelin sheath of the neuronal trajectories (oligodendrocytes).

Microglia/macrophages are equipped with a battery of pattern recognition receptors that detect pathogen-associated molecules (PAMPs) such as lipopolysaccharide (LPS) from bacterial infections or danger-associated molecular patterns (DAMPs) such as ATP [7, 34]. Activation of microglia/macrophages stimulates the release of IL-1β in a two-step process: the first is the stimulation of toll-like receptor 4 (TLR4) by LPS, leading to accumulation of cytoplasmic pro-IL-1β, and the second is the ATP-dependent stimulation of P2X7Rs, promoting nucleotide-binding, leucine-rich repeat, pyrin domain containing 3 (NLRP3) inflammasome-mediated caspase-1 activation and the consecutive secretion of IL-1β [28, 35]. Caspase-1 generates IL-1β from its precursor molecule pro-IL-1β by enzymatic decomposition.

## Pain as a sensory quality; P2X7Rs and chronic pain

**Fig. 1:**
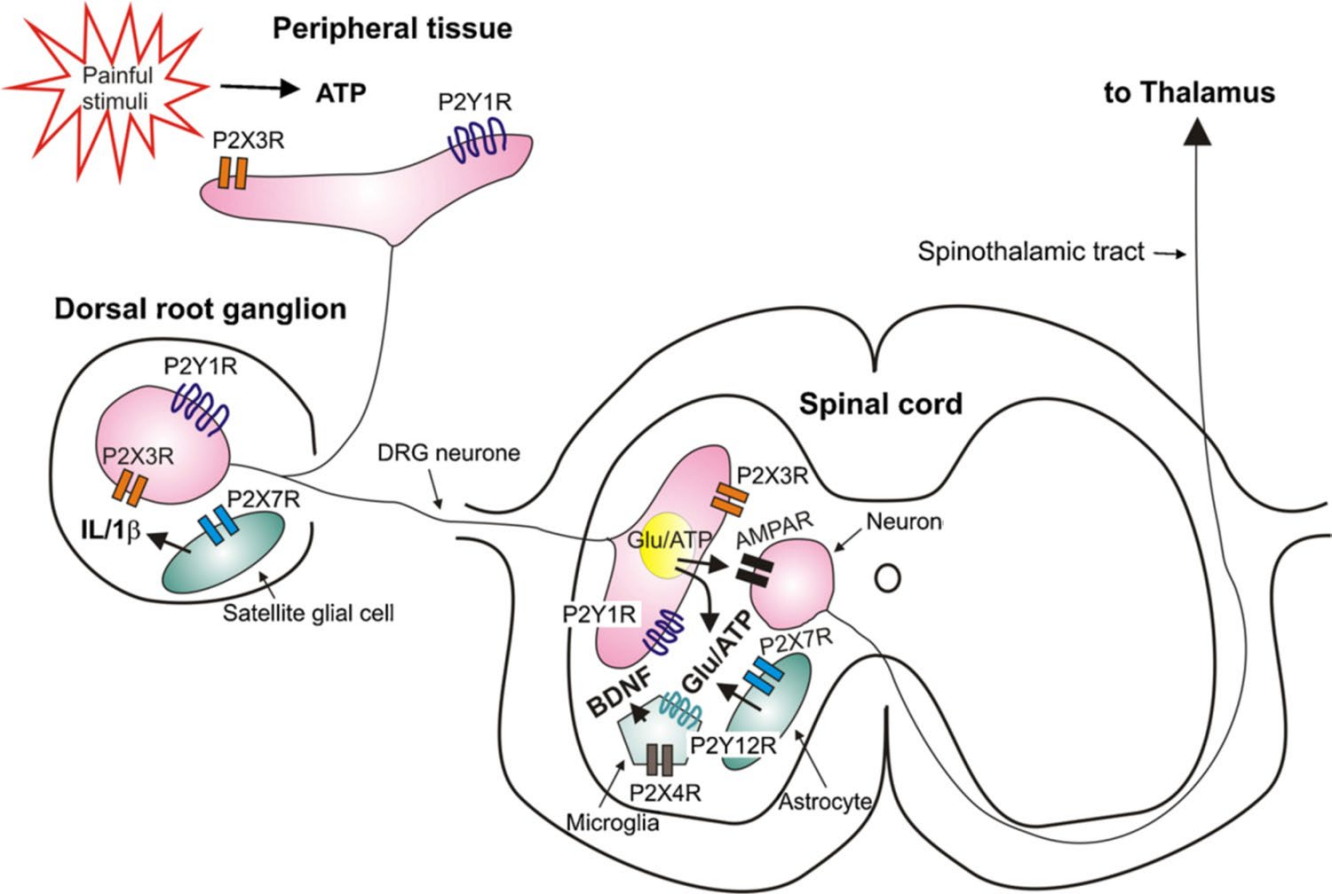
Schematic diagram of the first- and second-order pain neurons. The peripheral and central terminals of a dorsal root ganglion (DRG) neuron as well as its cell body contain P2X3 and P2Y1Rs. ATP released by various pathways from peripheral tissues or visceral organs may stimulate pain-inducing P2X3Rs and analgesia-inducing P2Y1Rs. Glutamate and ATP are sensory neurotransmitters in the synapse formed by the central axon terminals of DRG neurons and the spinothalamic neuron conducting the ascending information to the thalamus. Neuronal P2X3Rs in the dorsal root spinal horn increase the release of neurotransmitter glutamate. Satellite glial cells of the DRG and astrocytes of the dorsal root of the spinal cord possess P2X7Rs, which may release upon activation by ATP interleukin-1β (IL-1β) and the gliotransmitters glutamate/ATP. Microglial cells in the spinal cord dorsal horn are endowed with P2X4Rs, whose activation leads to the secretion of brain derived neurotrophic factor (BDNF) involved in the generation of allodynia and hyperalgesia being the main hallmarks of neuropathic pain. R, receptor; Glu, glutamate. Note that P2X2Rs were omitted from the cell body of the spinothalamic neuron. Reproduced with permission from [84]

Acute pain signals tissue damage and has a high diagnostic value as well as leads to accelerated healing by, e.g., enforcing the immobilization/protection of afflicted extremities. However, chronic pain loses its diagnostic value and develops to an independent disease causing ungrounded and overt suffering to patients. P2X7Rs were described to be involved because of their fundamental participation in inflammation in all types of chronic pain diseases comprising an inflammatory component. We will discuss different pain modalities such as neuropathic (of peripheral and central origin), and inflammatory pain, primary headache, regulation of morphine analgesic tolerance, and their dependence on P2X7R stimulation. Because of an obligatory inflammatory component, all types of chronic pain states were shown to respond to the pharmacological blockade of P2X7Rs [36–38]. The various P2X and P2Y receptor-types involved in the mediation of pain at the levels of peripheral tissue, dorsal root ganglion, and spinal cord are shown in Fig. 1.

### Peripheral neuropathic and inflammatory pain

Neuropathic pain has the cardinal symptoms of spontaneous, continuous and paroxysmal pain, hypersensitivity to painful stimuli (lower threshold), and allodynia. It was found relatively early that selective P2X7R antagonists relieved neuropathic and inflammatory pain in various animal models of these diseases [39–42]. Peripheral nerve injury (transection of the tibial and common peroneal nerves) caused an increase of both P2X7R mRNA and protein in the spinal cord [42]. Double-labelling immunohistochemistry demonstrated that cells expressing P2X7R protein after nerve injury were predominantly microglia. The intrathecal application of the P2X7R antagonist A-438079 suppressed the development of mechanical hypersensitivity in the hind-paw, ipsilateral to nerve damage. The tight involvement of P2X7Rs in neuropathic pain was proven by threefold evidence: (1) Disruption of the *P2RX7* gene abolished neuropathic hypersensitivity in a partial nerve ligation model to both mechanical and thermal stimuli, while normal nociceptive processing was undisturbed [43]. (2) In P2X7R deficient mice, the LPS-induced release of IL-1β from the peritoneal lavage of macrophages was abrogated [43] and the intrathecal LPS-induced mechanical hypersensitivity in the hind-paws was blocked [44]. (3) Both P2X7R antagonists [39, 42, 43] and intrathecal delivery of a palmitoylated peptide targeting Y_382-384_ at the C-terminus of the P2X7R [45] alleviated neuropathic pain caused by spared nerve injury. Y_382-384_ is a putative phosphorylation site that gates the potentiation of the P2X7R in a Src kinase-dependent fashion [46].

### Central neuropathic pain

Damage to peripheral nerves is accompanied by central sensitization of nociceptive neurons in the rat medullary dorsal horn (MDH). Application of the inflammatory irritant mustard oil to the tooth pulp of rats increased the mechanosensitive field and decreased the mechanical activation threshold of MDH neurons, when their extracellular activity was recorded electrophysiologically [47]. While continuous intrathecal superfusion with ATP or the more potent although non-selective P2X7R agonist Dibenzoyl-ATP (Bz-ATP) facilitated these responses, the P2X7R antagonists Brilliant Blue G and oxidized ATP depressed them. Superfusion with the microglial blocker minocycline abolished the mustard oil-induced central sensitization suggesting that the P2X7Rs are localized at microglia.

Central neuropathic pain is commonly caused by lesions or disease of the central somatosensory system. Frequent causes of this type of pain are spinal cord injury and cerebrovascular lesions [48]. In both cases, an upregulation of P2X7Rs occurs subsequent to the pathological enhancement of ATP release into the surrounding tissue. Neural stem cell transplantation has been shown to inhibit glial cell proliferation and P2X7R-mediated neuropathic pain in spinal cord injury of rats [49]. Microencapsulated neural stem cells were found to be even more efficient than neural stem cells alone [50]. It is assumed that this is due to a replacement of damaged cells as well as to the secretion of neurotrophic growth factors, and improvement of the microenvironment enhancing nerve regeneration. Hence, it was concluded that neural stem cell transplantation is one potential option for relieving neuropathic pain mediated by P2X7Rs.

### Primary headache

Primary headache is a common neurological disorder including diverse forms ranging from neuropathic type pain to migraine. A common mechanism of this complex disease involves activation and sensitization of the trigeminal system. Orofacial pain following chronic constriction injury of the infraorbital nerve resulted in tactile allodynia/hyperalgesia in rats and upregulated TNF-α in the trigeminal sensory nuclear complex [51]. The P2X7R antagonist A-438079 inhibited both responses to nervous injury. In another experimental setting, the right trigeminal ganglion of rats was electrically stimulated or formalin was injected to the whisker pad of rats [52]. Brilliant Blue G did not mitigate the behavioral responses but attenuated the increase in c-fos (immediate early gene)-positive cells in the caudal trigeminal nucleus.

Nerve endings isolated from the rat trigeminal caudal nucleus were loaded with tritiated aspartic acid in order to model the endogenous glutamate secretion [53]. ATP/Bz-ATP stimulated the spontaneous release of [^3^H]-aspartate in a manner blocked by P2X7R antagonists. AMPA plus Bz-ATP caused a larger release than Bz-ATP alone and the AMPAR antagonist NBQX blocked this excess release. It was concluded that P2X7Rs expressed on glutamateric nerve terminals in the trigeminal caudal nucleus can mediate transmitter release by facilitating the activation of presynaptic AMPARs. However, it is noteworthy that although pinched of nerve terminals (crude synaptosomal preparations) are often thought to be purely neuronal, these preparations are not homogenous and contain also glial material, microsomes, and mitochondria [54]. Hence, P2X7Rs may be localized at the astrocytic impurity contained in the synaptosomal preparations [31].

R192Q Ca_V_2.1 knock-in-mice (KI) express voltage-gated Ca_V_2.1 Ca^2+^ channels containing α_1A_ subunits harboring the R192Q missense mutation that leads to familial hemiplegic migraine type 1 in patients [55]. When trigeminal ganglion cultures were prepared and grown from wild-type and KI mice, the P2X7R-IR was stronger in non-neuronal than neuronal cells in both preparations and in addition, KI cultures showed higher expression of P2X7R-IR than wild-type cultures [56]. It was assumed that ATP is mainly released from meningeal mast cells during migraine-induced neuroinflammation [57, 58]. This ATP caused via P2X7R stimulation large intracellular Ca^2+^ transients in glial cells of trigeminal cultures [57].

### Regulation of morphine analgesic tolerance by P2X7Rs

Multiple factors are known to be involved in morphine tolerance, including desensitization of opioid receptors and functional changes in glutamate receptors and transporters [59, 60]. More recently, it was found that repeated administration of morphine leads to an upregulation of the protein level of spinal P2X7Rs [61]. Intrathecal administration of Brilliant Blue G attenuated the loss of morphine analgesia, P2X7R upregulation, and microglial activation. RNA interference targeting of spinal P2X7Rs exhibited tolerance-attenuating effect similar to Brilliant Blue G. Src family kinase activation mediated by µ-receptors was identified as a key mechanistic step required for morphine potentiation of P2X7R function [46]. Although P2X7Rs are obligatorily coupled to IL-1β secretion, chronic morphine treatment appeared to increase the expression of IL-18 by spinal microglia [62]. This effect was blocked by pharmacological antagonists or siRNA targeting P2X7Rs and restored analgesic tolerance.

Similar to the spinal, also supraspinal P2X7Rs in the midbrain periaqueductal gray (MPG) of rats were upregulated by repetitive morphine treatment [63]. The selective P2X7R antagonist A-74003, or antisense oligodeoxynucleotide targeting of the P2X7R, restituted the mechanical pain threshold decreased during morphine tolerance. The development of morphine tolerance was markedly alleviated by intra-MPG injection of D-amino acid oxidase (a D-serine degrading enzyme) [64]. D-serine is a gliotransmitter released from astrocytes/microglia and is a co-agonist at NMDARs. It is assumed that P2X7Rs are involved in D-serine production/release from glial cells to allow full NMDAR function in the MPG participating in the supraspinal mechanism of morphine tolerance.

Morphine was also found to paradoxically prolong neuropathic pain in rats by amplifying spinal NLRP3 inflammasome activation and the associated release of IL-1β from spinal microglia [65]. Selective inhibition of this signaling platform was achieved by in vivo transfection with a novel Designer Receptor Exclusively Activated by Designer Drugs (DREADD) into microglia. Treatment with the DREADD-specific ligand clozapine-*N*-oxide reversed morphine-induced persistent sensitization and reset pain to normal.

### Genetic variation of *P2RX7* and pain

The assessment of abnormal tactile hypersensitivity in mice of different genetic backgrounds after spared nerve injury showed robust inter-strain differences [36, 66]. Using genome-wide linkage analyses, an association between mechanical allodynia and the Pro45Leu mutation of the mouse P2X7R was established; mouse-strains in which P2X7Rs have impaired pore formation showed less allodynia than mice with pore-forming allele. A number of gain- and loss-of-function SNPs in patients with diabetic peripheral neuropathic pain were associated with higher pain intensity scores in females but not males [67]. The meta-analysis of two cohorts indicated that the loss-of-function P2X7R-SNP coding for Arg270His exhibited less pain in multisite chronic pain and postoperative pain than the wild-type receptor [68].

## P2X7Rs at microglia

All previous experiments suggest that the critical role of the P2X7R in the enhanced nociceptive transmission during neuropathic (and also inflammatory) pain is associated with microglial activation and secretion of IL-1β in the dorsal horn of the spinal cord. Peripheral nerve injury (dissection of the tibial and common peroneal nerves) increased both P2X7R-mRNA and protein in the rat spinal cord [42]. Double labelling immunohistochemistry revealed that the cells involved were predominantly microglia in the dorsal horn.

Pharmacological blockade by intrathecal administration of the P2X7R antagonist A-438079 suppressed the development of mechanical hypersensitivity. Activation of P2X7Rs stimulated the rapid maturation and release of IL-1β from macrophages and microglial cells ([35]; see above). At the level of the spinal cord, blockade of IL-1Rs with the IL-1R antagonist IL-1ra caused reduced nociception in animal models of inflammation- and nerve injury-induced pain [69, 70]. Additionally, mice lacking genes for both IL-1α and IL-1β showed attenuated inflammatory and neuropathic pain responses relative to wild-type mice [71]. In fact, in a model of Complete Freund’s Adjuvant (CFA)-induced long-lasting inflammation of the hind-paw, the P2X7R antagonist A-839977 produced robust antihyperalgesia in wild-type mice, but this effect was absent in IL-1αβ knockout mice [72].

IL-1β is released from microglia, packed in extracellular vesicles of variable shape/size generated by outward blebbing of the microglial plasma membrane; P2X7R activation was found to be the initiating factor for blebbing [73, 74]. Microvesicles shed from microglia were increased in the cerebrospinal fluid and dorsal horn of the spinal cord after spinal nerve ligation in rats [75]. IL-1β was found to be significantly upregulated in microvesicles, and the paw withdrawal threshold/paw withdrawal latency increased following inhibition with short-hairpin (sh) RNA-IL-1β. Hence, the antagonism at P2X7Rs might decrease the release of IL-1β from microglia and in consequence the hypersensitivity to neuropathic pain. The importance of P2X7Rs in determining the pro- (M1 polarization) or anti-inflammatory (M2 polarization) profile of microglial cells was demonstrated in a rat model of neuropathic pain induced by chronic compression injury of the sciatic nerve [76]. Botulinum toxin type A injection to the hind-paw returned the decreased mechanical withdrawal threshold/thermal withdrawal latency in neuropathic rats to their normal level apparently because of changing the M1 polarization of microglia to an M2 polarization.

In addition to the already mentioned secretion of cytokines/chemokines from microglia by P2X7R activation, proteases, reactive oxygen (ROS), and nitrogen species as well as potentially excitotoxic neuro/gliotransmitters (glutamate/ATP) may also be released from microglia/astrocytes. It has been reported that in spinal astrocytes, P2X7R activation induced ROS production at least partially through NADPH oxidase [77]. The results reveal that P2X7Rs on spinal astrocytes increase ROS production, subsequently leading to IL-6 release. Further, intrathecal application of Bz-ATP induced robust biphasic spontaneous nociceptive behavior [78]. Pre-treatment with the P2X7R antagonistic A-438079 abolished both the first and second phases of this response, while ROS scavengers attenuated only the second phase.

In addition to mechanical damage to major peripheral nerves also diabetes mellitus (DM) is a frequent cause of neuropathic pain because of the developing diabetic microangiopathy. Streptozocin was injected to destroy pancreatic β-cells in order to generate a model of DM type I in rodents [36, 79]. Streptozocin-treated mice expressed increased P2X7R protein in the dorsal horn of the lumbar spinal cord, and P2X7R-immunoreactivity was co-localized with the microglial marker Iba1. When these mice were injected intrathecally with the P2X7R antagonist A-740003, or when P2X7R^−/−^ mice were rendered diabetic/neuropathic, an attenuated progression of mechanical allodynia was observed.

## P2X7Rs at Schwann cells and astrocytes

As mentioned above, neuropathic pain is due to the upregulation of P2X7Rs in microglia. However, axonal regeneration in peripheral nerves is heavily dependent on P2X7Rs localized at Schwann cells producing the myelin sheath of these nerves [80]. Although the speed of axonal regeneration was unaltered in P2X7 knockout mice, the KO nerves were morphologically different from wild-type nerves. Lack of P2X7Rs committed Schwann cells to a non-myelinating phenotype during development.

Alike Schwann cells, astrocyte-like satellite cells in sensory ganglia also possess P2X7Rs. Thus, it was suggested that ATP released from dorsal root ganglion (DRG) neurons may activate P2X7 receptors at satellite cells, which in consequence release TNF-α potentiating P2X3R-mediated responses at nearby neurons, and thereby upregulate pain sensation [81]. Surprisingly, another group of authors reported opposite results, in that P2X7Rs in DRG satellite cells tonically inhibited the expression of P2X3Rs in neurons and downregulate pain [62, 82]. This inhibitory effect was via ATP release from satellite cells and the stimulation of P2Y1Rs at neurons. The cause for the divergent results is not really known but it is clear that neurons and surrounding glial cells in vegetative ganglia communicate with each other via signaling molecules; this communication may have implications for the manifestation of chronic pain [83, 84].

Tetanic stimulation to the sciatic nerve in vivo produced long-lasting hyperalgesia and allodynia in rats [85]. This pain reaction depended on the undisturbed functioning of spinal astrocytes, because the selective astrocytic toxin fluorocitrate partially impeded the stimulation-induced reduction of the paw withdrawal latency. In perfect agreement with these findings, activation of glial cells was found to be causative for the induction of long-term potentiation (LTP) at spinal C-fiber synapses [86]. Monosynaptic C fiber-evoked excitatory postsynaptic currents (EPSCs) were recorded from layer I neurons in rat lumbar spinal cord slices. Combined activation of microglia and astrocytes by Bz-ATP or high frequency stimulation to the primary afferent C-fibers resulting in ATP release induced LTP in C-fiber inputs. The Bz-ATP induced LTP was abolished by the respective antagonists A-438079 and fluoroacetate, another astrocytic toxin. Collectively, these and additional data indicated that the combined activation of microglia and astrocytes triggered gliogenic LTP at C-fiber synapses with spinal lamina I neurons through the release of two glial cell products, D-serine and TNF-α.

The substantia gelatinosa (SG; layer II) of the spinal cord dorsal horn is an important area of sensory integration in the CNS, where incoming C and A fiber axons of the primary afferents innervate local interneurons, which process nociceptive information to ascending projection neurons originating in the layer I of the spinal cord [87, 88]. SG neurons were shown to be activated by ATP/Bz-ATP indirectly through the stimulation of P2X7Rs located at neighboring astrocytes [89]. The astrocyte-neuron communication was exerted by the signaling molecules glutamate and GABA. A similar indirect stimulation by P2X7Rs was documented also in the hippocampal CA1 area of the rat brain [89]. In agreement with these findings, P2X7R activation at astroglia has been reported to release glutamate [90, 91], GABA [92], and ATP itself [93].

The suggestion that substantia gelatinosa interneurons do not contain P2X7Rs is part of a more general hypothesis questioning the existence of P2X7Rs at neurons in general [31, 94, 95]. Whereas early results appeared to support the operation of P2X7Rs at neurons, more recently, glial P2X7Rs are increasingly considered as indirect causes of neuronal effects. Specific tools for P2X7Rs are of limited value in deciding this question because of the poor selectivity of pharmacological agonists, and the inherent failure of antibodies to differentiate between the large number of active and inactive splice variants, or gain-of-function and loss-of-function SNPs of the receptor. Moreover, the available *P2rx7* KO mice generated by pharmaceutical companies possess some splice variants, which evade functional inactivation [2, 3]. However, the recent selective genetic inactivation or transgenic fluorescent labelling of P2X7Rs in glial cells or neurons turned out to be helpful to differentiate between neuronal and glial P2X7Rs [96, 97]. Extensive characterization of the Tg(RP24-114E20P2X7451P-StrepHis-EGFP)Ani reporter mouse revealed dominant P2X7-EGFP protein expression in microglia, Bergmann glia, and oligodendrocytes, but not in neurons. These findings were confirmed by microglia- and oligodendrocyte-specific P2X7 deletion and a novel P2X7-specific nanobody [31, 97].

## P2X7R antagonists as therapeutic means

The P2X7R has been the most extensively investigated subtype for drug development, and numerous potent and selective, mainly allosteric, antagonists have been characterized in the last decades [98, 99]. The development of P2X7R antagonists was somewhat handicapped by the fact that a selective agonist is missing (Bz-ATP activates P2X1 and P2X3Rs with high potency), mouse, rat, and human orthologues exhibit often different sensitivities to agonists/antagonists, and the various in vitro test-systems (Ca^2+^ influx, Yo-Pro uptake, and membrane current measurements) show divergent results even when tested with the same P2X7R ligand [100].

The classic antagonist Brilliant Blue G (BBG) differentiates with high selectivity P2X7Rs from other P2XR subtypes, although it does block in relatively low concentrations voltage-sensitive sodium channels [101]. In addition, BBG had to be applied over a couple of days in order to develop its full antagonistic activity [102]. However, improved P2X7R antagonists with high selectivity and suitable pharmacokinetic profiles are in the meantime available ([98, 103]; see chemical structures in Fig. 2 of [99].

P2X7R antagonists block the release of inflammatory cytokines and have therefore pronounced anti-inflammatory properties. They have been investigated for their therapeutic effects in rheumatoid arthritis [104, 105] and Crohn disease [106, 107]. Nevertheless, the development of these antagonists for the above mentioned indications was terminated by various pharmacological companies due to low efficiency or possible side effects [34, 103].

Based on these rather negative experiences, the research teams of pharmacological companies turned their attention to centrally acting, blood–brain barrier permeable P2X7R antagonists, inhibiting the development of depressive-like reactions in laboratory animals induced by acute or chronic stress [108–110]. Based on rat and mouse experiments, it is assumed that several P2X7R antagonists with excellent pharmacokinetic profiles and subcutaneous applicability may improve major depression and bipolar disorder in humans [111].

## Conclusions

P2X7Rs are localized at microglia/astrocytes and thereby secrete inflammatory cellular products to initiate and maintain chronic pain of various origin. In consequence, the fundamental involvement of P2X7Rs in pain diseases require the ongoing characterization of selective small molecular antagonists of these receptors. Fortunately, a number of such molecules are already available both as peripheral and blood–brain barrier permeable entities and are reaching gradually increasing significance as possible therapeutic agents for chronic pain states.

## Data Availability

The datasets supporting the conclusions of this article are included in the reviewed original publications.
